# Family history of diabetes and risk of SARS‐COV‐2 in UK Biobank: A prospective cohort study

**DOI:** 10.1002/edm2.283

**Published:** 2021-07-11

**Authors:** Bhautesh Dinesh Jani, Barbara I. Nicholl, Peter Hanlon, Frances S. Mair, Jason MR. Gill, Stuart R. Gray, Carlos A. Celis‐Morales, Frederick K. Ho, Donald M. Lyall, Jana J. Anderson, Claire E. Hastie, Mark ES. Bailey, Hamish Foster, Jill P. Pell, Paul Welsh, Naveed Sattar

**Affiliations:** ^1^ General Practice and Primary Care Institute of Health and Wellbeing University of Glasgow Glasgow UK; ^2^ Institute of Cardiovascular and Medical Sciences BHF Glasgow Cardiovascular Research Centre University of Glasgow Glasgow UK; ^3^ Public Health Institute of Health and Wellbeing University of Glasgow Glasgow UK; ^4^ School of Life Sciences College of Medical, Veterinary and Life Sciences University of Glasgow Glasgow UK

**Keywords:** diabetes, family history, lifestyle, SARS‐CoV‐2

## Abstract

**Introduction:**

The aim of this study was to determine risk of being SARS‐CoV‐2 positive and severe infection (associated with hospitalization/mortality) in those with family history of diabetes.

**Methods:**

We used UK Biobank, an observational cohort recruited between 2006 and 2010. We compared the risk of being SARS‐CoV‐2 positive and severe infection for those with family history of diabetes (mother/father/sibling) against those without.

**Results:**

Of 401,268 participants in total, 13,331 tested positive for SARS‐CoV‐2 and 2282 had severe infection by end of January 2021. In unadjusted models, participants with ≥2 family members with diabetes were more likely to be SARS‐CoV‐2 positive (risk ratio‐RR 1.35; 95% confidence interval‐CI 1.24–1.47) and severe infection (RR 1.30; 95% CI 1.04–1.59), compared to those without. The excess risk of being tested positive for SARS‐CoV‐2 was attenuated but significant after adjusting for demographics, lifestyle factors, multimorbidity and presence of cardiometabolic conditions. The excess risk for severe infection was no longer significant after adjusting for demographics, lifestyle factors, multimorbidity and presence of cardiometabolic conditions, and was absent when excluding incident diabetes.

**Conclusion:**

The totality of the results suggests that good lifestyle and not developing incident diabetes may lessen risks of severe infections in people with a strong family of diabetes.

## Graphical abstract

Our aim was to determine risk of severe SARS‐CoV‐2 (infection associated with hospitalization/mortality) in those with family history of diabetes. Using UK Biobank data, the findings suggest that having ≥2 family members with diabetes is associated with higher risk of SARS‐CoV‐2 infection and severe infection, but that such risk may be attenuated by better lifestyle, and by avoiding development of diabetes.

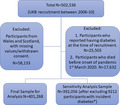

**Abbreviations**
BMI: Body mass index
CI: Confidence intervals
ICD: International Classification of Diseases
LTC: Long‐term conditions
RR: Risk ratio
SARS‐CoV‐2: Severe acute respiratory syndrome coronavirus 2
SD: Standard deviation

## 1 INTRODUCTION

People with existing health conditions are at higher risk of SARS‐CoV‐2 infection^1^ and related mortality, including people with diabetes.^2^ A meta‐analysis from 30 different studies found more than twice higher risk of SARS‐CoV‐2 disease and mortality among patients with diabetes.^3^

Family history (parental or sibling) of diabetes is a strong risk factor for type 2 diabetes, with stronger familiality (ie >one family member affected) being associated with higher risk.^4^, ^5^ Previous research has suggested that in addition to genetic risk, lifestyle‐related factors such as adiposity and dietary habits may explain some of this observed association.^4^, ^5^

The risk for SARS‐CoV‐2 infection and poor outcomes among those with family history of diabetes has not been previously investigated. Given the current uncertainty about the course and duration of the pandemic continues, and the prevalence of diabetes in many populations worldwide, the role of whether family history of diabetes in predisposition to damaging SARS‐CoV‐2 outcomes is important to know as diabetes can be prevented. We investigated the association of family history of diabetes with the risk of developing SARS‐CoV‐2 and severe SARS‐CoV‐2, and the potential role of demographic and lifestyle factors in such a link.

## 2 METHODS

### 2.1 Ethical approval

This study was conducted as part of UK Biobank project number 14151 and is covered by the generic ethics approval for UK Biobank studies from the NHS National Research Ethics Service (16/NW/0274). All participants gave written informed consent before enrolment in the study, which was conducted in accordance with the principles of the Declaration of Helsinki.

### 2.2 Study design

We used data from UK Biobank (UKB), a longitudinal cohort of 502,503 participants aged 37–73 recruited from the general population in England, Wales and Scotland between 2006 and 2010. SARS‐CoV‐2 test samples were collected and processed between 16th March 2020 and 31st January 2021. SARS‐CoV‐2 test results were provided by Public Health England (http://biobank.ndph.ox.ac.uk/ukb/exinfo.cgi?src=COVID19_tests). Participants who had died prior to the SARS‐CoV‐2 outbreak in the UK (01/03/2020) and who reported diabetes at the time of UKB study recruitment were excluded. Data were only available for participants in England. During the early study period of the ‘first wave’ of the outbreak in England, SARS‐CoV‐2 testing was highly selective and limited to only those participants who presented with severe symptoms.

### 2.3 Exposure variable

Family history of diabetes (mother/father/sibling) was reported at the time of recruitment and categorized into none, one, or two or more, according to the number of family members with a history of diabetes.

### 2.4 Outcome variables

Confirmed SARS‐CoV‐2 infection (≥one positive result) and severe SARS‐CoV‐2 infection (defined as testing positive during an inpatient hospital episode).

### 2.5 Sociodemographics, lifestyle and underlying health conditions

Age at recruitment was used as a continuous variable. Sex and ethnicity (categorized into ‘Asian or Asian British’, ‘Black or Black British’, ‘Chinese’, ‘Mixed’, ‘Other ethnic group’ or ‘White’ groups) were used as categorical variables. Townsend score, a measure of socioeconomic status based on participant postcode, was used as a continuous score.^6^ Smoking status self‐reported at the time of UKB study recruitment was categorized as never, previous or current. Body mass index (BMI) was measured by a study nurse at the time of UKB study recruitment and used as a continuous variable. Level of physical activity was defined as ‘none’, ‘low’, ‘medium’ or ‘high’ using Metabolic Equivalent of Task (MET) minutes per week scores based on the International Physical Activity Questionnaire (IPAQ) scoring protocol 2005. The physical and mental health conditions self‐reported by participants at the time of UKB study recruitment were organized into a list of 43 long‐term conditions (LTCs) based on our previously published literature.^7^, ^8^ Multimorbidity count was classified by LTC count into 0, 1, 2, 3 or ≥4. Presence of four cardiometabolic conditions (hypertension, diabetes, ischaemic heart disease and stroke) was ascertained via participant self‐report at the time of UKB study recruitment or hospitalization event (between UKB study recruitment to April 2019) with ICD‐10 codes as primary or secondary discharge diagnoses (incident).

### 2.6 Statistical analysis

We used Poisson regression models with robust standard errors to test for cross‐sectional associations between family history of diabetes and SARS‐CoV‐2 infection or severe SARS‐CoV‐2. Unadjusted estimates were presented followed by stepwise adjustments. The models were first adjusted for sociodemographic variables (age, sex, Townsend score and ethnicity), then for lifestyle variables (smoking status, BMI and physical activity levels), and finally for presence of cardiovascular conditions at the time of study recruitment (hypertension, ischaemic heart disease and stroke), incident admissions for cardiometabolic conditions (hypertension, diabetes, ischaemic heart disease and stroke) and multimorbidity count.

### 2.7 Sensitivity analysis

The main analysis was repeated for the subset of participants who were tested positive for SARS‐CoV‐2, with the risk of severe SARS‐CoV‐2 as the outcome variable. The above analysis was also repeated after excluding participants who were diagnosed with diabetes (based on hospitalization records) since the study recruitment to the onset of COVID pandemic.

All analyses were carried out using R version 3.6.1, using the ‘glm’ function and ‘quasipoisson’ formula.

## 3 RESULTS

**FIGURE 1:**
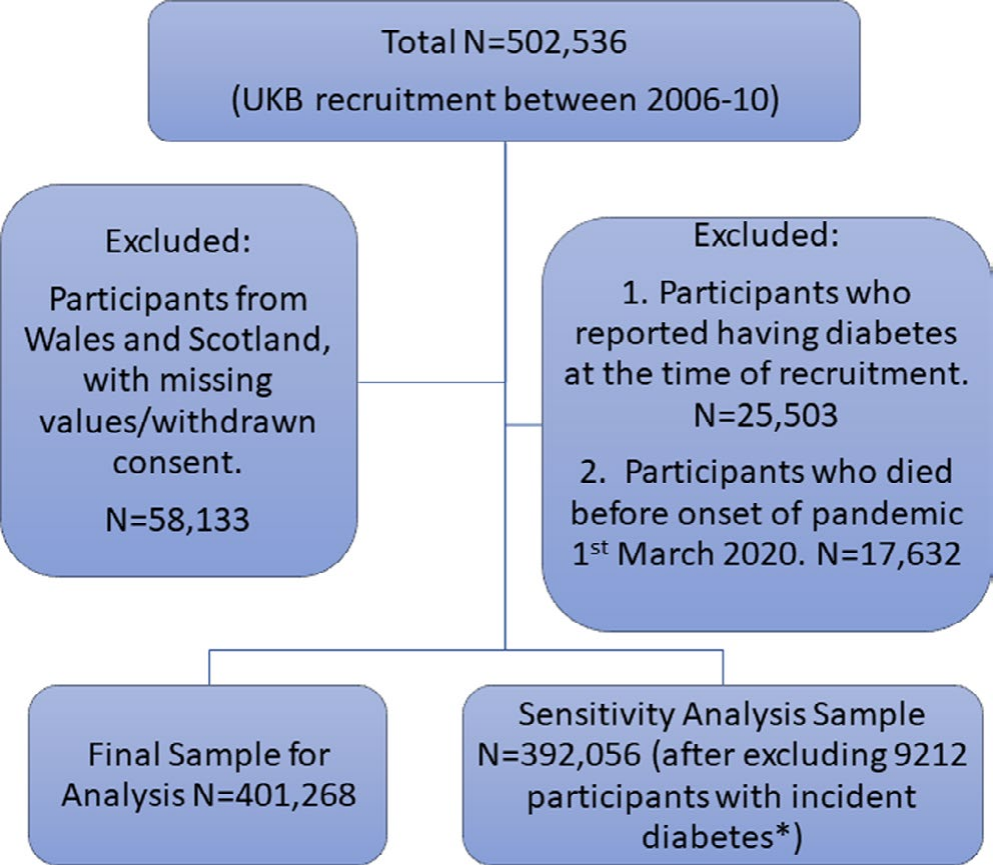
Flow chart of study participants. *Incident diabetes, participants who were diagnosed with diabetes (based on hospitalization records) since the study recruitment to the onset of COVID pandemic; UKB, UK Biobank

After exclusions noted above, 401,268 were included for analyses (please see Figure 1). On recruitment, 70,427 participants (17.5%) reported having one family member with diabetes, and 12,443 (3.1%) reported having two or more family members with diabetes (Table 1).

**TABLE 1:** Family history (none/one/two or more) of diabetes and risk of SARS‐CoV‐2 infection/severity in UK Biobank. *N* ^a^ = 401268

	Exposure variables categories	HbA1c Mean (SD)	Unadjusted	Model 1 (adjusted for demographics‐ age, sex, ethnicity and deprivation	Model 2 (model 1 plus lifestyle‐ smoking, BMI and physical activity)	Model 3 (model 2 plus cardiometabolic conditions only incident diabetes and MM count)
Outcome One: 13331 SARS‐CoV−2 positive						
Family history of Diabetes	None 10214/318398	35 (4.4)	1	1	1	1
	One 2577/70427	35.6 (5.0)	**1.14 (1.09–1.19); p < 0.01**	**1.07 (1.03–1.12); *p* < 0.01**	**1.04 (1.00–1.09); *p* = 0.03**	**1.04 (1.00–1.09); *p* = 0.04**
	Two or more 540/12443	36.7 (5.6)	**1.35 (1.24–1.47); *p* < 0.01**	**1.17 (1.07–1.27); *p* < 0.01**	**1.11 (1.02–1.21); *p* = 0.01**	**1.10 (1.00–1.19); *p* = 0.04**
Outcome Two: 2282 Severe^b^ SARS‐CoV−2 infection						
Family history of Diabetes	None 1771/318398	35 (4.4)	1	1	1	1
	One 421/70427	35.6 (5.0)	1.07 (0.96–1.19); *p* = 0.18	1.08 (0.97–1.20); *p* = 0.16	1.03 (0.92–1.15); *p* = 0.52	1.01 (0.91–1.13); *p* = 0.74
	Two or more 90/12443	36.7 (5.6)	**1.30 (1.04–1.59); *p* < 0.01**	1.20 (0.96–1.48); *p* = 0.09	1.06 (0.84–1.32); *p* = 0.57	1.01 (0.80–1.25); p = 0.93

Note

Model 1: Adjusted for age, sex, deprivation score and ethnicity; Model 2: Adjusted for model 1 plus body mass index, physical activity levels and smoking; Model 3: Adjusted for model 2 plus multimorbidity count and presence of diabetes (incident), hypertension (baseline and incident), stroke (baseline and incident), ischaemic heart disease (baseline and incident).

Abbreviuations: CI, confidence intervals; RR, risk ratio; SD, standard deviation.

^a^ Participants who died before the onset of SARS‐CoV‐2 pandemic and those who reported having diabetes at baseline were excluded.

^b^ Severe SARS‐CoV‐2 defined as participants who tested positive during an inpatient hospital episode.

Results in bold suggest statistical significance (*p* < 0.05).

In total, 13,331 (3.32%) tested positive for SARS‐CoV‐2 and 2282 (0.6%) participants had severe SARS‐CoV‐2 (ie infection detected in hospital or through death). The proportion of study sample testing positive of SARS‐CoV‐2 infection was 3.2%, 3.65% and 4.33% respectively for those with no, one and two or more family members with diabetes. Participants with at least one family member with diabetes had higher average HbA1c than participants with no family history (see Table 1). In the unadjusted model, participants with two or more family members with diabetes were more likely to test positive for SARS‐CoV‐2 infection (risk ratio (RR) 1.35; 95% confidence intervals (CI) 1.24–1.47) and have severe infection (RR 1.30; 95% CI 1.04–1.59) than participants with no family history. The association with risk of testing positive was attenuated after adjusting for sociodemographics (RR 1.17; 95 CI 1.07–1.27), and additionally for lifestyle factors (RR 1.11; 95% CI 1.02–1.21) and multimorbidity (RR 1.10; 95% 1.00–1.19). (Table 1).

The proportion of the study sample with severe SARS‐CoV‐2 was 0.56%, 0.60% and 0.72% respectively for those with no, one and two or more family members with diabetes. Participants with two or more family members with diabetes were more likely to have severe SARS‐CoV‐2 infection (RR 1.30; 95% CI 1.04–1.59) in unadjusted model. The association was no longer significant after adjusting for demographics, lifestyle factors and underlying health.

### 3.1 Sensitivity analysis

Table 2 shows the results of association between family history of diabetes and risk of severe SARS‐CoV‐2 only for the subset of participants who were tested positive for SARS‐CoV‐2. The results show that there was no significant association between family history of diabetes and severe SARS‐CoV‐2 in the subset who tested positive. Table 3 shows the results of sensitivity analysis after excluding participants who were diagnosed with diabetes (based on hospitalization records) since the study recruitment to the onset of COVID pandemic. In the sensitivity analysis, there was significant association between family history of diabetes and testing positive for SARS‐CoV‐2 infection; however, risk was attenuated after adjusting for demographics, lifestyle factors and underlying health. In this group, there was no association between family history of diabetes of severe COVID‐19 infections.

**TABLE 2:** Sensitivity Analysis: Family history (none/one/two or more) of diabetes and risk of SARS‐CoV‐2 severity in UK Biobank in subset of participants who were positive for SARS‐CoV‐2. *N* = 13331

	Exposure variables categories	HbA1c Mean (SD)	Unadjusted	Model 1 (adjusted for demographics‐ age, sex, ethnicity and deprivation	Model 2 (model 1 plus lifestyle‐ smoking, BMI and physical activity)	Model 3 (model 2 plus cardiometabolic conditions only incident diabetes and MM count)
Outcome: 2282 Severe^a^ SARS‐CoV−2 infection						
Family history of Diabetes	None 1771/10214	34.9 (4.7)	1	1	1	1
	One 421/2577	35.6 (5.8)	0.94 (0.85–1.04); *p* < 0.01	1.01 (0.91–1.12); *p* = 0.80	0.99 (0.90–1.10); *p* = 0.86	0.98 (0.89–1.08); *p* = 0.74
	Two 90/540	36.4 (5.3)	0.96 (0.79–1.16); *p* = 0.24	1.03 (0.84–1.25); *p* = 0.73	0.98 (0.80–1.20); *p* = 0.86	0.98 (0.80–1.19); *p* = 0.86

Note

Model 1: Adjusted for age, sex, deprivation score and ethnicity; Model 2: Adjusted for model 1 plus body mass index, physical activity levels and smoking; Model 3: Adjusted for model 2 plus multimorbidity count and presence of diabetes (incident), hypertension (baseline and incident), stroke (baseline and incident), ischaemic heart disease (baseline and incident). Participants who died before the onset of SARS‐CoV‐2 pandemic and those who reported having diabetes at baseline were excluded.

Abbreviations: CI, confidence intervals; RR, risk ratio; SD, standard deviation.

^a^ Severe SARS‐CoV‐2 defined as participants who tested positive during an inpatient hospital episode.

Results in bold suggest statistical significance (*p* < 0.05).

**TABLE 3:** Sensitivity Analysis: Family history (none/one/two or more) of diabetes and risk of SARS‐CoV‐2 infection/severity in UK Biobank after excluding those with incident diabetes. *N* = 392056^a^

	Exposure variables categories	HbA1c Mean (SD)	Unadjusted	Model 1 (adjusted for demographics‐ age, sex, ethnicity and deprivation	Model 2 (model 1 plus lifestyle‐ smoking, BMI and physical activity)	Model 3 (model 2 plus cardiometabolic conditions only incident diabetes and MM count)
Outcome One: 12851 SARS‐CoV−2 positive						
Family history of Diabetes	None 9888/312356	34.8 (4.1)	1	1	1	1
	One 2463/68046	35.4 (4.5)	**1.14 (1.09–1.19); *p* < 0.01**	**1.08 (1.03–1.12); *p* < 0.01**	**1.05 (1.01–1.10); *p* = 0.02**	**1.05 (1.01–1.10); *p* = 0.02**
	Two 500/11654	36.2 (4.8)	**1.35 (1.24–1.48); *p* < 0.01**	**1.18 (1.07–1.28); *p* < 0.01**	**1.13 (1.03–1.23); *p* < 0.01**	**1.12 (1.03–1.23); *p* = 0.01**
Outcome Two: 2108 Severe^b^ SARS‐CoV‐2 infection						
Family history of Diabetes	None 1649/312356	34.8 (4.1)	1	1	1	1
	One 388/68046	35.4 (4.5)	1.08 (0.97–1.20); *p* = 0.17	1.08 (0.97–1.21); *p* = 0.16	1.03 (0.92–1.15); *p* = 0.61	1.02 (0.96–1.17; *p* = 0.51
	Two 71/11654	36.2 (4.8)	1.15 (0.90–1.45); *p* = 0.24	1.06 (0.83–1.34); *p* = 0.62	0.96 (0.75–1.21); *p* = 0.75	0.95 (0.72–1.26; *p* = 0.71

Note

Model 1: Adjusted for age, sex, deprivation score and ethnicity; Model 2: Adjusted for model 1 plus body mass index, physical activity levels and smoking; Model 3: Adjusted for model 2 plus multimorbidity count and presence of hypertension (baseline and incident), stroke (baseline and incident), ischaemic heart disease (baseline and incident).

Abbreviations: CI, confidence intervals; RR, risk ratio; SD, standard deviation

^a^ Participants who died before the onset of SARS‐CoV‐2 pandemic and those who reported having diabetes at baseline and incident were excluded.

^b^ Severe SARS‐CoV‐2 defined as participants who tested positive during an inpatient hospital episode.

Results in bold suggest statistical significance (*p* < 0.05).

## 4 DISCUSSION

Given diabetes is a strong prognostic factor for SARS‐CoV‐2 severity and mortality,^9^ relatives of such patients may be rightly concerned that they are also at higher risk. The present analyses have shown this to be the case with a 35% higher chance of a positive SARS‐CoV‐2 test and a 30% higher chance of severe SARS‐CoV‐2 infection in those with two first‐degree relatives with diabetes. However, it appears as if this risk may be mitigated by better lifestyles which also lessen the risk of comorbidity. Lifestyle factors such as smoking, BMI and physical activity are likely to influence the risk of COVID‐19 and/or associated complications as evidenced by previous studies. A separate study using UK Biobank data found higher risk of severe SARS‐CoV‐2 with physical inactivity, smoking and obesity but not heavy alcohol consumption.^10^ In addition, relationship between unhealthy lifestyle and severe disease risk was dose dependent. Another study by the COVID‐19 host genetics initiative found similar association between high BMI, smoking and physical activity and higher risk severe COVID‐19 illness using mendelian randomization approach.^11^ Hopkinson et al. found that people who smoke were at higher risk of developing symptomatic COIVD‐19, based on data from 2.4 million UK users of the Zoe COVID‐19 symptom study.^12^ A study in United States found that patients with COVID‐19 who were physically inactive had a higher risk of hospitalization, ICU admission and mortality, with some evidence of dose‐response relationship.^13^

We accept some limitations. UK Biobank participants are relatively healthier and more affluent than general UK population although there is some evidence that risk factor associations with health outcomes are generalizable.^14^ The work did not differentiate between type 1 and type 2 diabetes, though most with two family members are likely to have Type 2 diabetes. The data on family history of diabetes and lifestyle behaviour were collected at the time of study recruitment (2006–2010) and have not been updated since. Also, we could not fully adjust for all incident diabetes; only admissions for diabetes were adjusted for. In addition, information on lifestyle and underlying health was based on self‐report and these data were collected more than 10 years ago. During the early study period of the ‘first wave’ of the outbreak in England, SARS‐CoV‐2 testing was highly selective and limited to only those participants who presented with severe symptoms. There was no association found between family history of diabetes and severe SARS‐CoV‐2 risk when analysis was limited to those who tested positive to reduce the bias due to selective testing. That said, this sensitivity analysis by no means excludes bias as those initially tested for SARS‐CoV‐2 in the UK had more severe symptoms and it is impossible to ascertain whether viral loads (and so symptoms and testing) differed dependent on family history of diabetes. Of course, other unmeasured factors could also have biased the chances of testing. Therefore, we believe our main findings hold relevance to those with family histories of diabetes.

The association of being SARS‐CoV‐2 positive with family history of diabetes may partially reflect both a strong association between excess body weight and SARS‐CoV‐2 infection or related mortality (as recently summarized in a UK Governmental report)^15^ and the strong social factors (ie lower socioeconomic status) linked to SARS‐CoV‐2 infection that overlap with diabetes risk. The risk attenuation after adjustment for lifestyle factors provides some reassurance that healthy lifestyle may not only attenuate risk of diabetes in those with strong family histories but may also attenuate risk of SARS‐CoV‐2 disease and severe complications. The sensitivity analyses also point in similar direction. However, this attenuation in risk is likely related to lifestyle over many years and so our findings do not necessarily demonstrate that short‐term change in lifestyle would immediately alter the risk of SARS‐CoV‐2 infection, although such short‐term changes can prevent diabetes. Our findings may stimulate those with diabetes family histories to take more interest in preventive efforts.

## CONFLICT OF INTEREST

NS reports personal fees from Amgen, AstraZeneca, Eli Lilly, Merck Sharp & Dohme, Novo Nordisk, Pfizer and Sanofi, and grants and personal fees from Boehringer Ingelheim, outside the submitted work. BDJ, BIN, PH, FSM, JMRG, SRG, CAC‐M, FKH, DML, JJA, CEH, MESB, HF, JPP and PW declare no conflict of interest.

## AUTHOR CONTRIBUTIONS

The idea for this paper was generated by NS and BDJ. The analyses were done by BDJ. All other authors gave input to the writing and revision of the manuscript. NS is guarantor, and takes responsibility for the contents of the article.

## DATA AVAILABILITY STATEMENT

The data used in this study are available via UK Biobank (https://www.ukbiobank.ac.uk/), subject to necessary approvals.
